# Fritillariae Thunbergii Bulbus: Traditional Uses, Phytochemistry, Pharmacodynamics, Pharmacokinetics and Toxicity

**DOI:** 10.3390/ijms20071667

**Published:** 2019-04-03

**Authors:** Hong Li, Andrew Hung, Mingdi Li, Angela Wei Hong Yang

**Affiliations:** 1 School of Health and Biomedical Sciences, RMIT University, Bundoora, Victoria 3083, Australia; s3640112@student.rmit.edu.au (H.L.); s3335658@student.rmit.edu.au (M.L.); 2 School of Science, RMIT University, Melbourne, Victoria 3001, Australia; andrew.hung@rmit.edu.au

**Keywords:** natural product, traditional medicine, complementary and alternative medicine, herbal medicine, review

## Abstract

Fritillariae Thunbergii Bulbus (FTB) has been widely used as an antitussive herb for thousands of years in China. However, FTB’s traditional uses, chemical compounds and pharmacological activities have not been systematically reviewed. This study aimed to review its traditional uses, phytochemistry, pharmacodynamics, pharmacokinetics and toxicity. We searched the Encyclopedia of Traditional Chinese Medicine to explore the historical records which indicate that it acts to clear heat, resolve phlegm, relieve cough, remove toxicity and disperse abscesses and nodules. We searched 11 databases to identify potential phytochemical or pharmacological studies. Characteristics of its chemical constituents, pharmacological effects, pharmacokinetic and toxicity were descriptively summarized. A total of 9706 studies were identified and 83 of them were included. As a result, 134 chemical constituents were identified, including 26 alkaloids, 29 compounds found in essential oils, 13 diterpenoids, two carbohydrates, two sterols, 18 amino acids, six nucleosides, four nucleobases, four fatty acids, three lignans, and 27 elements. Thirteen pharmacological effects of FTB were identified, including anti-cancer, tracheobronchial relaxation, antitussive, expectorant, anti-muscarinic, anti-inflammation, anti-thyroid, regulation of blood rheology, antiulcer, anti-diarrhea, pain suppression, antioxidation and neuroprotection. These pharmacological activities may be mainly attributed to the alkaloids in FTB. Further phytochemical, pharmacological and network pharmacological studies are recommended.

## 1. Introduction

Fritillariae Thunbergii Bulbus (FTB) is also known as Zhe bei mu or Xiang bei mu in Chinese, Setsubaimo in Japanese and Jeolpaemo in Korean [1]. Fritillariae Thunbergii belongs to the Fritillariae (Liliaceae) family and FTB has been widely used as an antitussive herb for thousands of years in China [2]. Fritillariae Thunbergii is widely cultivated in the south-eastern coastal, south-central and eastern areas of China, such as Zhejiang, Jiangsu, Anhui and Hunan provinces [2]. FTB is a significant traditional Chinese herb with bitter and cold properties, entering the Lung and Heart channels [2]. It is generally prescribed as one of the ingredients of herbal formula, such as Danggui Beimu Kushen Wan (Chinese Angelica, Fritillaria and Flavescent Sophora Pill; DBKW) in traditional and contemporary clinical practice [3,4]. Recently, investigating potential treatments from ancient records has been employed as a strategy to direct novel drug discovery [5]. Modern studies that are recorded in the Zhong Yao Da Ci Dian (Great Compendium of Chinese Medicines) [6] and Zhong Hua Ben Cao (Chinese Pharmacopoeia) [7] indicated that the pharmacological effects of FTB include antitussive, tracheobronchial relaxation, anti-muscarinic, expectorant and pain suppression with a wide range of chemical constituents, such as peimine and peiminine. With the development of modern methodological approaches, an increasing number of new chemical constituents (e.g., frithunbol A and frithunbol B) [8], and pharmacological effects (e.g., anti-cancer activity [9] and neuroprotection [8]) have been found in FTB. Therefore, there is a need to thoroughly review FTB from traditional and modern perspectives to assist further drug discovery. This study aimed to review traditional uses, phytochemical, pharmacodynamic, pharmacokinetic and toxicity characteristics of FTB and provide comprehensive and evidence-based information to practitioners and researchers.

## 2. Traditional Uses of FTB

The Zhong Hua Yi Dian (Encyclopedia of Traditional Chinese Medicine; ZHYD) CD-ROM, containing 1156 ancient books, was searched to identify potential classic literature of FTB [10]. FTB was first documented in the book Ben Cao Hui Yan (BCHY, Discourse on Herbal Medicine, NI Zhumo, 1624, Ming Dynasty), with its actions described as breaking blood and detoxication. In addition, five classic books, including Ben Cao Zheng Yao (Evidence of Materia Medica, LI Zhongzi, 1673, Qing dynasty), Ben Jing Feng Yuan (Doctrine of Origin, ZHANG Lu, 1695, Qing dynasty), Ben Cao Gang Mu Shi Yi (Supplement to the Compendium of Materia Medica, ZHAO Xuemin, 1765, Qing dynasty), Ben Cao Bian Du (Simple Materia Medica, ZHANG Bingcheng, 1887, Qing dynasty) and Ben Cao Zheng Yi (Merits of Herbal Medicine, ZHANG Shanlei, 1920, Republic of China), specified that the unique actions of FTB were to clear the Lung and calm the Liver, disperse the Lung-Qi to relieve depression, clear the Heart and reduce the heat, which were not found in other Fritillariae species, such as Fritillariae Cirrhosae Bulbus (Chuan bei mu) and Bulbus Bolbostemmatis Rhizoma (Tu Bei Mu). In summary, FTB acts to clear heat, resolve phlegm, relieve cough, remove toxicity and disperse abscesses and nodules. A number of herbal formulas containing FTB were identified. Supplementary file 1 presents some example formulas containing FTB as an ingredient in ZHYD. Herbal ingredients in Supplementary file 1 are listed as Chinese pinyin name as per the nomenclature list of commonly used Chinese herbal medicines published by the Chinese Medicine Board of Australia [11]. Today, some of them are continuously used by practitioners in evidence-based Chinese medicine clinical practice, such as DBKW, Guang Bi Shu Zhan Tang (Throat Tuberculosis Decoction), Xiao Lei Wan (Eliminate Scrofula Pill), Xing Su Yin (Apricot Kernel and Perilla Leaf Decoction), Qing Jin Ning Sou Tang (Clear the Lung and Stop Cough Decoction), Tuo Li Pai Nong Tang (Support the Interior and Drain Pus Decoction), Ren Shen Bai Du San (Ginseng Powder to Overcome Toxin) and Xian Fang Huo Ming Yin (Sublime Formula for Sustaining Life).

In terms of the indications of FTB, Ben Cao Zheng (Orthodox Materia Medica, ZHANG Jiebin, 1624, Ming dynasty) recorded that it could be used for a number of conditions, including lung abscess, lung atrophy, cough, dyspnea, hematemesis, epistaxis, hematochezia, hematuria, jaundice, difficult urination, pharyngitis, hemorrhoid, anal fistula, acute mastitis, scrofula and any furuncles, carbuncle, abscess and other inflammation and swelling. Ben Cao Cong Xin (New Revised Materia Medica, WU Yiluo, 1751, Qing dynasty) claimed that FTB could be utilized for patients with flu induced by wind-phlegm. Ben Cao Gang Mu Shi Yi provided further descriptions that the indications of FTB could be extended to any respiratory disorders which are due to wind-fire and phlegm. As shown in Supplementary file 1, those formulas had been widely used in various disciplines along with the development of Chinese medicine, including otorhinolaryngology, ophthalmology, external medicine, gynecology, obstetrics, galactophore and infectious diseases.

## 3. Modern Exploration of FTB

The following seven English and four Chinese electronic databases were searched from their respective inceptions to 2 November 2018: PubMed, EMBASE, CINAHL, Science Direct, Scopus, ProQuest, Allied and Complementary Medicine Database, CNKI, CQVIP, Wanfangdata and CBM. Keywords used to identify potential studies included: Zhe bei mu and its English, Latin, Japanese, Korean, botanical and pharmaceutical names and their synonyms. The reference list of review articles was screened manually for potential studies.

Original experimental studies on phytochemistry and pharmacology, or the studies containing phytochemical or pharmacological tests, published in English or Chinese, were considered for inclusion, regardless of the type of experimental studies (e.g., in vivo and in vitro). The studies were included if they investigated a single raw herb with species and specific medicinal parts of FTB listed in the Pharmacopoeia of the People’s Republic of China. The literature was excluded if they are review articles or do not include original phytochemical or pharmacological studies, or did not use a raw herb with appropriate species or medicinal parts of FTB, or directly used chemical compounds instead of a raw herb as the research material, or published in a non-English or non-Chinese language.

One reviewer (HL) screened the searched literature and extracted the following data to a predesigned Excel form: Compound name, molecular formula, molecular weight, molecular structure and analysis methods for phytochemical studies; and compound name, study type, characteristics of sample, interventions, duration, outcome measures, main results for pharmacological studies. Corresponding molecular structures were further searched in the PubChem database to obtain the PubChem CID/SID number. The molecular structures that could not be found in PubChem were drawn manually using the software ChemDraw. All included studies were divided into two groups: Studies reporting a voucher number (SRV) and studies not reporting voucher number (SNRV). A voucher specimen number is one of the most significant requirements for phytological studies, since it ensures the reliability of botanical taxon and the repeatability of published studies [12,13]. The data was checked and confirmed by the second reviewer (ML). When any discrepancies between the two reviewers occurred, they were discussed with the third party (AY) to resolve the issues. A descriptive summary was performed to obtain the characteristics of the extracted data.

**Figure 1:**
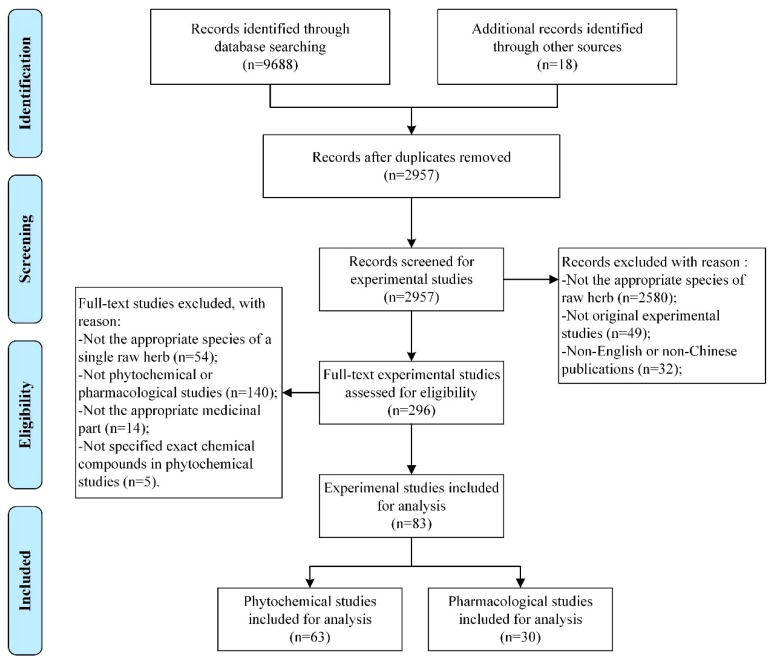
Flowchart of the selection process of Fritillariae Thunbergii Bulbus studies.

A total of 9706 experimental studies were identified adhering to the search strategies mentioned above. Eighty-three studies meeting the inclusion criteria were included for analysis, including 63 phytochemical studies [8,14,15,16,17,18,19,20,21,22,23,24,25,26,27,28,29,30,31,32,33,34,35,36,37,38,39,40,41,42,43,44,45,46,47,48,49,50,51,52,53,54,55,56,57,58,59,60,61,62,63,64,65,66,67,68,69,70,71,72,73,74,75] and 30 pharmacological studies [8,9,17,27,33,45,49,56,59,73,75,76,77,78,79,80,81,82,83,84,85,86,87,88,89,90,91,92,93,94], as 10 studies reported both phytochemical and pharmacological effects of FTB [8,17,27,33,45,49,56,59,73,75]. Figure 1 details the selection process of included studies.

### 3.1. Phytochemistry of FTB

A total of 134 chemical constituents were identified, including 26 alkaloids (A1–A26), 29 compounds found in essential oils (B1–B29), 13 diterpenoids (C1–C13), two carbohydrates (D1–D2), two sterols (E1–E2), 18 amino acids (F1–F18), six nucleosides (G1–G6), four nucleobases (H1–H4), four fatty acids (I1–I4), three lignans (J1–J3), and 27 elements (K1–K27). Table 1 details the characteristics of chemical constituents isolated from FTB. 

**Figure 2:**
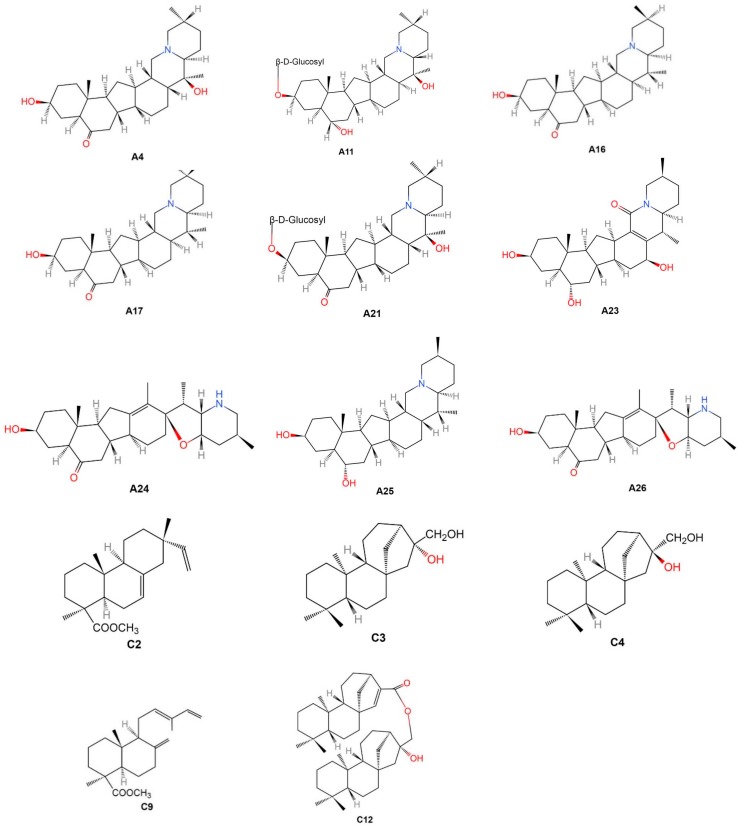
Molecular structures of the 14 chemical constituents of Fritillariae Thunbergii Bulbus. Note: A4: Zhebeinone; A11: Zhebeininoside; A16: Eduardine; A17: Zhebeirine; A21: Zhebeinone-3-β-d-glucoside; A23: Frithunbol A; A24: Frithunbol B; A25: Eduardinine; A26: 3β-hydroxy-5α-jervanin-12-en-6-one; C2: Isopimaran-19-oic acid, methyl ester; C3: Ent-kauran-16β, 17-diol; C4: Ent-kauran-16α, 17-diol; C9: Trans-comminic acid, methyl ester; C12: Ent-16β-hydroxy-kauran-17-yl ent-kaur-15-en-17-oate.

Within 63 included phytochemical studies, there are 17 SRV (47 constituents isolated) [8,14,21,26,33,38,43,44,50,56,58,60,62,63,70,71,72] and 46 SNRV (118 constituents isolated) [15,16,17,18,19,20,22,23,24,25,26,27,28,29,30,31,32,34,35,36,37,39,40,41,42,45,46,47,48,49,51,52,53,54,55,57,59,61,64,65,66,69,73,74,75]. Thirty-one chemical constituents were identified in both SRV and SNRV groups, including nine alkaloids (A1, A2, A5, A6, A7, A9, A11, A14 and A15), four nucleosides (G1-G4), one nucleobases (I1), and 17 elements (K1, K2, K6-K12, K14, K16, K17, K19, K20, K22, K25 and K27). In terms of the molecular structures, 117 chemical constituents were found in the PubChem database that refers to the corresponding PubChem CID/SID, however, 17 constituents were not available in PubChem. Amount of the 17 chemical components, 14 of the molecular structures (A4, A11, A16, A17, A27, A30-A33, C2-C4, C9 and C12) could be found in the included studies and three of them (A10, A22 and D1) could not be found anywhere. Details of the 14 molecular structures were presented in Figure 2.

#### 3.1.1. Alkaloids

Current studies mainly focused on the alkaloids which are the fundamental components of FTB [34]. Among 26 isolated alkaloids in FTB, 0.080% of peimine (A1) and peiminine (A2) used for identification and quality control as listed in the Pharmacopeia of People’s Republic of China [2]. These two compounds were first isolated in 1960 using the paper partition chromatography method [46] and then they were consistently found by over 40 research teams using more than 20 different methods from 1960 to 2018 [8,17,18,20,21,22,23,24,26,27,28,29,31,32,33,36,37,38,39,41,42,44,45,47,48,49,51,56,57,59,60,63,70,72,73,75]. The in vivo and in vitro experimental studies identified the biological activities of peimine (A1) and peiminine (A2), such as anti-cancer activity [49], tracheobronchial relaxation [17], anti-muscarinic activity [75], anti-inflammatory activity [33,88], and pain suppression [88], which indicates they may have great potential in clinical practice. In addition, some isomers were also reported, including peimine (A1) [33], zhebeinine (A3) [68] and isoverticine (A7) [56] with the same molecular formula C_27_H_45_NO_3_, peiminine (A2) [21], zhebeinone (A4) [65] and suchengbeisine (A8) [33] with the same molecular formula C_27_H_43_NO_3_, ebeiedine (A5) [33], puqiedine (A15) [72], N-demethylpuqietinon (A19) [73] and eduardinine (A25) [8] with the same molecular formula C_27_H_45_NO_2_, ebeiedinone (A6) [56], puqiedinone (A14) [72], eduardine (A16) [66] and zhebeirine (A17) [66] with the same molecular formula C_27_H_43_NO_2_, and frithunbol B (A24) [8] and 3β-hydroxy-5α-jervanin-12-en-6-one (A26) [8] with the same molecular formula C_27_H_42_NO_3_. Another two new steroidal alkaloids were isolated and identified in 2018 [8]. They are the colorless gum called frithunbol A (A23) and frithunbol B (A24) with the structures 5α-cevanin-13-ene-3β,6α,16β-triol-18-one and 3β-hydroxy-5α-jerv-12-en-6-one respectively, presented in Figure 2. Frithunbol B (A24) could reduce nitric oxide (NO) in vitro and it may possess neuroprotective activity [8].

#### 3.1.2. Compounds from Essential Oils

A total of 29 compounds found in essential oils in FTB were investigated in two recent experimental studies using the gas chromatography–mass spectrometry method. One study reported 15 of them, including δ-elemene (B1), δ-selinene (B2), tetradecanoic acid (B3), pentadecanoic acid (B4), hexadecanoic acid, methyl ester (B5), 9-hexadecenoic acid (B6), n-hexadecanoic acid (B7), kaur-15-ene (B8), heptadecanoic acid (B9), kaurene (B10), 9,12-octadecadienoic acid (Z,Z)-, methyl ester (B11), 9-tetradecenal, (Z)- (B12), 9,12-octadecadienoic acid, methyl ester, (E, E)- (B13), oleic acid (B14), and linoleic acid, ethyl ester (B15) [16]. Among these 15 constituents, n-hexadecanoic acid (B7), 9,12-octadecadienoic acid, methyl ester, (E, E)- (B13), and oleic acid (B14) were the three primary compounds found in essential oils in FTB accounting for 53.46%, 26.96% and 9.34% respectively. Another study also found hexadecanoic acid, methyl ester (B5), and reported another 14 chemical compounds, including butylated hydroxytoluene (B16), L-(+)-Ascorbic acid 2,6-dihexadecanoate (B17), ethyl 9-hexadecenoate (B18), hexadecanoic acid, ethyl ester (B19), 1H-naphtho [2,1-B] pyran, 3-ethenyldodecahydro-3,4a,7,7,10a-pentamethyl- (B20), kaur-16-ene (B21), 9,11-octadecadienoic acid, methyl ester, (E, E)- (B22), 9,12-octadecadienoic acid (B23), linoleic acid, ethyl ester (B24), octadecanoic acid, ethyl ester (B25), 2(1H)-phenanthrenone, 3,4,4a,4b,5,6,7,8,10,10a-decahydro-1,1,4a,7,7-pentamethyl, [4aR-(4a.α., 4b.β.,10a.β.)]- (B26), 3-methyleneandrostan-17-ol (B27), androst-4-En-3-one, 17-hydroxy-, (17.β.) (B28), and podocarp-7-en-3.β.-ol, 13.β.-methyl-13-vinyl- (B29) [25]. The latter study stated the proportion of four main compounds found in essential oils: Linoleic acid, ethyl ester (B24; 36.93%), kaur-16-ene (B21; 22.85%), hexadecanoic acid, methyl ester (B5; 10.67%), and 3-methyleneandrostan-17-ol (B27; 10.59%). However, the proportion of hexadecanoic acid, methyl ester (B5) in the former study was only 0.6% [25]. It is worth noting that different compounds from essential oils with different proportions from FTB have been identified, due to the different isolation methods. The former study soaked FTB powders for 12 h and distilled it for 10 h [16] whereas the soak time and distillation time in the later study were one hour and five hours respectively [25]. Further studies should consider utilizing the appropriate isolation method to achieve selective isolation of target compounds. However, at the current stage, there is no solid evidence to identify which isolation method is better.

#### 3.1.3. Diterpenoids

Two studies conducted by the same Japanese research team in 1984 focused on the diterpenoids in FTB [34,35]. Thirteen diterpenoids were identified using the nuclear magnetic resonance methods (1HNMR and 13CNMR), including isopimaran-19-ol (C1), isopimaran-19-oic acid, methyl ester (C2), ent-kauran-16β, 17-diol (C3), ent-kauran-16α, 17-diol (C4), ent-16β, 17-epoxy-kaurane (C5), ent-16α-methoxy-kauran-17-ol (C6), ent-kaur-15-en-17-ol (C7), trans-communol (C8), trans-comminic acid, methyl ester (C9), ent-17-norkauran-16-one (C10), ent-15β,16-epoxy-kauran-17-ol (C11), ent-16β-hydroxy-kauran-17-yl ent-kaur-15-en-17-oate (C12), and ent-(16S)-atisan-13, 17-oxide (C13) [34,35]. However, no further studies, after 1984, were found on the investigation of the diterpenoids or the specific diterpenoids identified from FTB. As diterpenoids in other Chinese herbs may perform diverse biological activities, such as anti-cancer activity [95], anti-inflammatory activity [96] and antioxidative activity [97], further studies which focus attention on diterpenoids in FTB may be important.

#### 3.1.4. Nucleosides

Three studies identified six nucleosides from FTB using three different methods (LC-ESI-MSn [70], HPLC-UV-ESI/MS [70] and HPLC [64,71]), including uridine (G1), guanosine (G2), adenosine (G3), thymidine (G4), cytidine (G5), and inosine (G6). One of the studies highlighted that the content of nucleosides in FTB could be used as an index for the identification and quality control of FTB [64]. However, to date, none of the included studies evaluated the pharmacological activities of nucleosides isolated from FTB. As the basic building-block of DNA and RNA, nucleosides from other plants possess many biological activities, such as anti-cancer activity [98], antibacterial activity [99], and antiviral activity [100]. It is worth investigating nucleosides in FTB for novel drug discovery.

#### 3.1.5. Elements

A total of 27 elements were found for identification and quality control purposes [14,15,40,43,52,53,55,58,61,62,74]. Eight of them are trace elements that may have vital biological activities in the human body, including cobalt, copper, iron, manganese, molybdenum, sulfur, selenium and zinc [14,15,40,43,52,54,55,58,61,62,74]. Another six trace elements without any known biological activities were identified in FTB, including arsenic, cadmium, chromium, mercury, nickel and lead [14,15,40,43,55,58,61,62,74]. They have toxic activities that may damage the lipids, proteins, enzymes and DNA even in low dose [101]. All the toxic elements in the included studies were under the maximum limited level listed in the Pharmacopeia of the People’s Republic of China [14,15,40,43,52,53,55,58,61,62,74].

#### 3.1.6. Other Constituents

Apart from above chemical components, 33 chemical compounds belonging to six categories were isolated and identified, which are used for identification, as well as quality control, including two carbihydrates (β-d-glucose4-1β-d-galactose (D1) and sucrose (D2)) [19], two sterols (β-sitosterol (E1) and daucosterol (E2)) [68], 18 amino acids (glycine (F1), leucine (F2), methionine (F3), tyrosine (F4), histidine (F5), threonine (F6), alanine (F7), isoleucine (F8), tryptophan (F9), cystine (F10), lysine (F11), aspartic acid (F12), valine (F13), phenylalanine (F14), proline (F15), serine (F16), glutamic acid (F17) and arginine (F18)) [64], four nucleobases (adenine (H1), hypoxanthine (H2), uracil (H3) and thymine (H4)) [64,70], four fatty acids (vernolic acid (I1), 2-monopalmitin (I2), 13(R)-hydroxy-octadeca-(9Z,11E,15Z)-trien-oic acid (I3) and picropodophyllotoxin (I4)) [67,73], and three lgnans (octahydrocurcumin (J1), zhebeiresinol (J2) and sauriol B (J3)) [30,73].

### 3.2. Pharmacology of FTB

**Figure 3:**
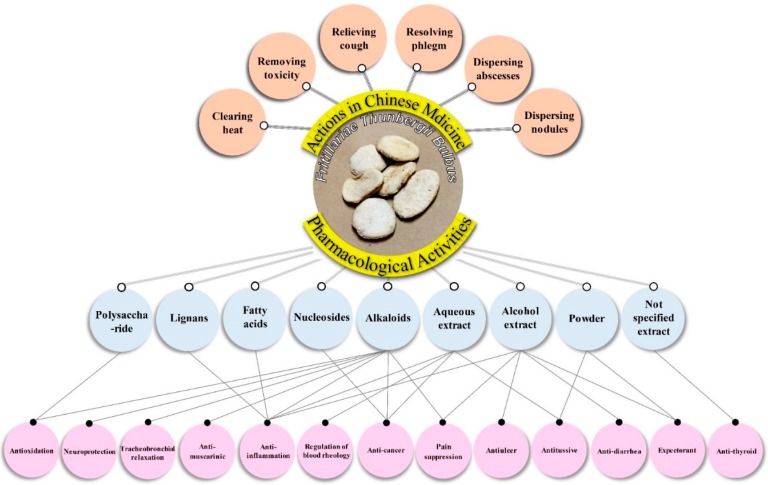
Multiple pharmacological effects of Fritillariae Thunbergii Bulbus.

There are 23 studies focused on the pharmacological effects of FTB, including anti-cancer, tracheobronchial relaxation, antitussive, expectorant, anti-muscarinic, anti-inflammation, anti-thyroid, regulation of blood rheology, antiulcer, anti-diarrhea, pain suppression, antioxidation and neuroprotection (Figure 3). Characteristics of the 23 included pharmacological effects are summarized in Table 2.

#### 3.2.1. Anti-Cancer Effect

Cancer is one of the most life-threatening conditions with high morbidity and mortality in the world [102]. However, the therapeutic effects of conventional medicine are unsatisfactory, due to many adverse effects, such as fatigue, nausea and vomiting [103]. Increasingly, natural products have been identified with anti-cancer activity, which may be associated with better therapeutic effects and less adverse effects [104,105]. Four included studies indicated that FTB had anti-cancer effect [9,49,80,91]. Three of them investigated the multidrug resistance (MDR) reversal activity of FTB on human lung adenocarcinoma parental cells A549 [80], human hepatocellular carcinoma cell line HepG2 [9], and human breast carcinoma cells MCF-7 [49]. Li and colleagues reported that the total alkaloids of FTB may have the MDR reversal activity on A549/cisplatin (A549/DDP) cells [80]. Specifically, the 50% inhibitory concentration (IC50) of FTB extract in vitro was 141 ± 5 mg/L (A549 cells) and 298 ± 22 mg/L (A549/DDP cells) while the IC50 DDP plus FTB (9 mg/L) and DDP alone on A549/DDP cells was 14.06 ± 3.72 mg/L and 0.79 ± 0.14 mg/L respectively after 72-h intervention. The reversal fold (RF) of FTB to DDP on A549/DDP cells was 17.80, higher than other reversal agents, such as cyclosporine A (10.16) and tetrandrine (14.05). The tumor inhibitory rate of DDP plus FTB (2 mg/kg) was 67.4%, higher than DDP alone (49.9%). The expressions of MDR1 mRNA and P-glycoprotein (P-gp) were significantly decreased by FTB extract. Similar results on the expression of P-gp were also reported by another research team in China [9]. They used the Calcein-AM Kit to identify potential P-gp inhibitors. The results showed that alkaloids and nucleoside in FTB significantly restricted the efflux activity of P-gp in a dosage-dependent manner. Furthermore, Tong specified that peimine (A1) and peiminine (A2) were the reversal agents in FTB that reversed the MDR of adriamycin or paclitaxel in MCF7/A cells. The RF of peimine (A1) plus adriamycin or paclitaxel and peiminine (A2) plus adriamycin or paclitaxel was 8.17, 4.57, 3.30 and 3.73 respectively after 48-h treatment. In addition, direct anti-cancer activity was also detected by in vitro and In vivo studies in one included study [91]. The researchers believed that the FTB aqueous extract (75 mg/mL) inhibited the human lung adenocarcinoma parental cells LM2 with dosage dependence and the apoptosis rate was increased over time (24 h: 5.7 ± 0.91%; 48 h: 11.7 ± 1.07%). In vivo studies revealed that the tumor inhibitory rate of FTB aqueous extract (1.6 g/kg) was 33.33%. Concurrently, the same dose of FTB aqueous extract significantly reduced the number of metastases in mice (5.7 ± 1.72), compared to the control groups (7.3 ± 2.2) [91]. FTB, especially peimine (A1) and peiminine (A2), seems to have both direct and indirect anti-cancer effects evidenced by inhibiting the growth of tumor cells, as well as reversing the MDR of conventional chemotherapy drugs, such as DDP, adriamycin and paclitaxel [9,49,80,91]. Nonetheless, existing evidence is not sufficient to elucidate its mechanisms of actions on lung, liver or breast cancer. Therefore, more in vitro and In vivo studies are needed on lung, liver, breast and other cancers to thoroughly investigate its direct and indirect anti-cancer activity in the future.

#### 3.2.2. Tracheobronchial Relaxation

Two included studies investigated the tracheobronchial relaxation activity of FTB extract in isolated bronchial and tracheal rings of rats [17,56]. Chan claimed that peimine (A1), peiminine (A2), ebeiedine (A5), and puqietinone (A13) extracted from FTB relaxed the tracheobronch in vitro with 6.9 ± 0.1, 6.4 ± 0.12, 6.3 ± 0.09 and 5.7 ± 0.07 bronchi pD_2_ values respectively and 6.9 ± 0.12, 6.8 ± 0.18, 6.5 ± 0.08 and 6.0 ± 0.16 tracheas pD_2_ values [17]. pD_2_ values, also called pEC_50_, is the negative logarithm of the half maximal effective concentration (EC_50_) and if the pEC_50_ values are larger, the drugs are more effective [106]. Thus, the ranking order of effects within these compounds are peimine (A1) ≥ peiminine (A2) > ebeiedine (A5) > puqietinone (A13) [17]. The researcher believed that the mechanisms of action of tracheobronchial relaxation activity of FTB may be related to activation of Ca^2+^-activated K^+^ channels, since peimine (A1), peiminine (A2) and ebeiedine (A5) inhibited Ca^2+^-induced contraction (concentration: 3 mM Ca^2+^) during the study [17]. Another recent study also investigated the pD_2_ values of FTB on tracheobronchial relaxation activity [56]. The results indicated that the total alkaloids of FTB have tracheobronchial relaxation activity with 6.66 ± 0.06 bronchi pD_2_ values and 6.71 ± 0.03 tracheas pD_2_ values, significantly higher than the crude water extract (4.7 ± 0.07 bronchi pD_2_ values and 4.63 ± 0.14 tracheas pD2 values) [56]. These two studies indicated that FTB could have tracheobronchial relaxation activity, due to its alkaloids, such as peimine (A1), peiminine (A2), ebeiedine (A5) and puqietinone (A13) [17,56]. However, more solid evidence is required to identify the best treatment dosage of these compounds, as well as the effects of other alkaloids on tracheobronchial relaxation activity.

#### 3.2.3. Antitussive Effect

Two In vivo studies were conducted by the same research team to identify the antitussive effect of FTB in guinea pigs [89,90]. Both of them indicated that the frequency of cough was significantly reduced (15 ± 7.6/5 min) and the remission period was prolonged (73.65 ± 43.02 t/s) to five minutes after oral administration of micro powders of FTB, compared to the purified water control group (24.2 ± 10.75/5 min and 46.99 ± 12.02 t/s respectively) [89,90]. Another study compared the antitussive effects of FTB in mice harvested in four different places in China [27]. The results indicated that a high dose of FTB aqueous extract (0.104 g/kg) harvested in all four different places significantly reduced the frequency of cough (*p* < 0.01) and increased the remission period (*p* < 0.05) [27]. As an important antitussive herb in ancient China, micro powders and aqueous extract of FTB appeared to have antitussive activity in experimental studies.

#### 3.2.4. Expectorant Effect

Two studies described FTB’s expectorant effect [86,89]. Yan and colleagues evaluated the expectorant effects of FTB fine powders via the phenol red expectoration test [89]. The results indicated that FTB fine powders significantly reduced the amount of phlegm secretion (phenol red contraction: 1.048 ± 0.09 mg/L), compared to the blank control group (phenol red contraction: 0.691 ± 0.059 mg/L) (mean difference 0.36, 95% confidence interval 0.29–0.42) [89]. The other study assessed the amount of phlegm secretion using a percentage (per hour secretion before and after administration) [86]. The result showed that FTB dramatically reduced the amount of phlegm secretion (203.6%, larger than the standard 170% which indicates effective treatment), compared to the control group (101.2%) [86]. These two studies showed that the fine powders and alcohol extract of FTB produce the expectorant effect evidenced by decreasing the amount of phlegm secretion [86,89]. However, the active compounds related to this effect are still unknown. Further studies should investigate the active chemical constituents in FTB, as well as possible mechanisms of actions related to the expectorant effect.

#### 3.2.5. Anti-inflammation

Anti-inflammation is one of the most essential pharmacological effects of FTB, mentioned in five included studies, three in vitro studies [33,73,88] and two In vivo studies [87,93]. Three chemical compounds isolated from FTB, including peimine (A1), ebeiedine (A5) and suchengbeisine (A8), could reduce the expression and production of MUC5AC mucin (common mucin expressed in the airway surface epithelium) in human pulmonary mucoepidermoid NCI-H292 cells [33]. Peimine (A1) was able to block the Kv1.3 ion channel [88]. The Kv1.3 inhibitor is commonly believed to have the anti-inflammatory ability, blocking the human T lymphocytes-induced immune responses [88,107,108]. Another five chemical compounds, including isoverticine (A7), puqiedine (A15), N-demethylpuqietinone (A19), 2-monopalmitin (I2), and zhebeiresinol (J2) reduced the expression of NF-κB level in the human embryonic kidney cells HEK293 [73]. In vivo studies revealed that the aqueous extract of FTB inhibited the abnormal immune response by relieving the inflammation and over the proliferation of fibroblasts of the prostate, as well as reducing the serum level of NO in the mice model with immunological chronic prostatitis/chronic pelvic pain syndrome [87]. Two animal studies reported that the alcohol extract of FTB relieved the swelling of ear and foot plantar induced by xylene and carrageenin respectively in mice [93]. With its anti-inflammatory activities, FTB could be used for many inflammatory disorders, such as pulmonary or tracheobronchial inflammatory disorders, lump or masses [87,93]. Future studies should focus on mechanisms of actions of the active compounds mentioned in these publications, including peimine (A1), ebeiedine (A5) and suchengbeisine (A8), isoverticine (A7), puqiedine (A15), N-demethylpuqietinone (A19), 2-monopalmitin (I2), and zhebeiresinol (J2) for anti-inflammatory activity.

#### 3.2.6. Pain Suppression

FTB was found to be a potential pain reliever in two included studies [88,92]. Peimine (A1) extracted from FTB inhibited the Nav1.7 ion channel, which could be a possible mechanism of action of pain suppression [88]. Nav1.7, commonly found in the peripheral nervous system, is one number of the sodium channel family encoded by the SCN9A gene [109,110,111]. Blocking this channel may lead to inherited gene mutations and subsequently lead to pain sensory loss [112]. The alcohol extract of FTB reduced the frequency of mouse writhing (acetic acid induced pain test) and prolonged the remission period of tail-flick latency (thermal stimulus pain test) in mice [92]. The in vitro and in vivo studies mentioned above presented some evidence relevant to the pain suppressive activity of FTB. However, due to limited evidence, it is difficult to thoroughly explain the mechanisms of this action. Further studies could consider using peimine (A1) as an active compound for pain relief in in vitro and in vivo studies.

#### 3.2.7. Antioxidation

Recent studies indicated that many herbs, including FTB have an antioxidative effect [84,85]. The total alkaloids extracted from FTB has strong antioxidative capacity that was reflected in the data of 2,2-diphenyl-1-picrylhydrazyl (DPPH) radical scavenging activity (EC_50_: 5.5 mg/mL), 2,2′-azino-bis (3-ethylbenzothiazoline-6-sulphonic acid) radical scavenging activity (EC_50_: 0.3 mg/mL) and ferric reducing capacity test (118.2 mg ascorbic acid equivalent per 100 g) [85]. The polysaccharide extracted from FTB also has an antioxidative effect [84]. Specifically, the DPPH scavenging ratio of FTB was 21.46%. The reducing capacity and total antioxidative capacity of FTB polysaccharide (1 mg/mL) were evaluated using the absorption spectroscopy method (700 nm) with 0.51 and 0.39 absorbance respectively [84]. Notwithstanding the fact that both of the in vitro studies indicated that not only total alkaloids, but also polysaccharide in FTB have a strong antioxidative capacity, in vivo studies are needed to support in vitro findings in future. In addition, identifying the active compounds related to this pharmacological effect is required.

#### 3.2.8. Other Pharmacological Effects

FTB also had other pharmacological effects, including anti-ulcer, anti-muscarinic, anti-thyroid, regulation of blood rheological properties, anti-diarrhea and neuroprotection [8,75,78,82,92,93,94]. The alcohol extract of FTB could directly inhibit water immersion-induced or hydrochloric acid-induced gastric ulcer [92], as well as an acetic acid-induced oral ulcer in rats [94]. Three alkaloids, including peimine (A1), peiminine (A2) and puqietinone (A13), could significantly increase the cAMP level in the HEK293 cells transfected with muscarinic M2 receptor [75]. Anti-thyroid activity was detected, evidenced by reduced the serum levels of T_3_, T_4_, cAMP and cGMP, and increasing hypoxia tolerance in hyperthyroidic mice [82]. FTB was able to decrease whole blood viscosity, inhibit erythrocyte aggregation and increase erythrocyte deformability in rats, which means it could regulate blood rheological properties [78]. In addition, the alcohol extract of FTB significantly decreased the frequency of diarrhea induced by castor oil or Fan xie ye (*Folium Sennae*) [93]. The NO level in lipopolysaccharide-activated BV-2 cells was inhibited by ebeiedinone (A6) (IC_50_: 11.45 μM), suchengbeisine (A8) (IC50: 18.02 μM) and frithunbol B (A24) (IC_50_: 16.35 μM) [8]. Moreover, the compound 3β-hydroxy-5α-jervanin-12-en-6-one (A26) significantly increased the nerve growth factor level (134.81 ± 3.66%) in C6 glioma cells [8]. In vivo and in vitro studies demonstrated that FTB may have a series of pharmacological effects. However, evidence for each pharmacological effect of FTB is limited. More in vitro and in vivo studies are needed to elucidate the possible mechanisms of actions of each effect.

### 3.3. Pharmacokinetics of FTB

Only six included studies of this review provided information on the pharmacokinetics of FTB’s chemical compounds, such as peimine (A1), peiminine (A2) and peimisine (A9) [45,49,59,76,77,83]. The experimental study indicated that peiminine (A2), extracted by 70% or 90% ethyl alcohol, could penetrate excised rat skin using ultraviolet radiation testing method [83]. The penetration rates were similar in both concentrations of solvent. Although the penetration amount of peiminine (A2) in different concentrations of ethyl alcohol was similar and increased over time, the percutaneous permeability coefficient was limited and low in both groups. A sensitive, rapid and maneuverable method, LC-TRAP-MS, was developed to identify whether the nine alkaloids extracted from FTB could be absorbed by rats in plasma after oral administration [45]. The study claimed that six compounds, including peimine (A1), peiminine (A2), ebeiedinone (A6), isoverticine (A7), peimisine (A9), and puqiedinone (A14) were detected in plasma whereas three of them, including ebeiedine (A5), zhebeininoside (A11) and puqiedine (A15) were not. Furthermore, the parameters of pharmacokinetics of peimine (A1), peiminine (A2) and peimisine (A9) were investigated in four studies, including maximum concentration (C_max_), elimination half-life (T_1/2_), time to reach C_max_ (T_max_), oral clearance (CL/F), volume distribution/bioavailability (V/F), area under the concentration-time curve (AUC) from zero to t and from zero to infinite time [49,59,76,77]. Table 3 summarizes the pharmacokinetic parameters of peimine (A1), peiminine (A2) and peimisine (A9) in these four studies. 

One of these four studies compared the differences of pharmacokinetics, tissue distribution and excretion between male and female rats [77]. Table 3 shows that in this study, the index (Cmax, Tmax, AUC0-t and AUC0-∞) of male rats was significantly higher than the female’s while the CL/F and V/F statistics were lower, which indicates that the elimination of peimine and peiminine in male rat plasma was slower than the female’s (*p* < 0.05). In contrast, the results also showed that the cumulative excretion of peimine (A1) and peiminine (A2) in male rat urine were significantly higher than that in female. Additionally, peimine (A1) and peiminine (A2) were found in various tissues of rats, including liver, heart, spleen, kidney, lung, uterus, ovary, brain, testis, muscle and skin [77]. The concentrations of both alkaloids in male tissues were significantly higher than female’s, except for muscle and skin. On the other hand, the pharmacokinetic parameters in Tong’s study were substantially lower than those in the other three studies, and this may be due to its very low administration dose (0.45 g/kg) [49]. Current pharmacokinetic studies of FTB are mainly focused on the absorption, distribution and excretion of peimine (A1), peiminine (A2) and some other alkaloids in rats. The examination of other chemical compounds will assist in understanding the pharmacokinetic profile of FTB. Further research should consider comprehensively investigating the pharmacokinetics of other chemical components listed in Table 1.

### 3.4. Toxicity of FTB

FTB is considered a non-toxic or low toxic herb for the past several thousands of years with a recommended dose of 5−10 g (equivalent to 83–167 mg/kg body weight) for adults [2]. Recently, three included studies were published which are relevant to the toxicity of FTB [79,81,92]. An in vivo study using 110 male Wistar rats indicated that the FTB water extract (10 g/kg, ig, for seven days) did not increase the creatine kinase and lactate dehydrogenase levels compared to the saline group [79]. The histopathological examination in this study showed that the basic structure of heart tissue and cardiac muscle fibers were normal, compared to the saline group. This revealed that the extract of FTB did not induce cardiotoxicity in rats. Nonetheless, irregular cell nuclear arrangement and nonuniform cytoplasm staining were found in the FTB group.

Another two studies investigated the median lethal dose (LD_50_) of oral intake FTB extract on 40 and 60 mice respectively via the acute toxicity test for seven [92] and 14 days [81] respectively. The results showed that the LD_50_ was 12.2 ± 2.2 g/kg [92] and 53 mg/kg [81] on mice respectively. Due to insufficient information, it is difficult to interpret the dramatic difference of the LD_50_ between these two studies. Acute toxic symptoms were observed, such as tremors, restlessness, drowsiness, asthma, convulsions, urinary incontinence, and tail-erecting reaction, and the cause of death could be pulmonary venous pleonaemia and respiratory failure [81,92]. A sub-chronic toxicity test was conducted on 120 rats after oral-intake of 1 or 3 mg/kg FTB extract for 26 weeks [81]. The findings indicated that the safe dose of FTB extract could be 1 mg/kg without any toxicity symptoms whereas tremor and reduction of spontaneous motor activities were found in the 3 mg/kg group. Furthermore, hematology, blood biochemistry, as well as weight and histology of rats’ organ were observed without any significant changes within 26 weeks [81].

The recommended dose listed in the Pharmacopeia of the People’s Republic of China seems to be safe, as the aqueous extract of FTB in recommended dose could be 0.0415–0.0835 mg/kg (0.05% extraction efficiency), which is approximately 10–25 times lower than 1 mg/kg [81]. Nonetheless, some challenges should not be neglected, such as the tremendous difference of LD_50_ in two different studies and the adverse effects found in the animal studies. Therefore, more experimental and clinical toxicity studies are needed, with a focus on the estimation of LD_50_, as well as the adverse effects of FTB at both low and high doses.

## 4. Conclusions

It is worth noting that this study was the first to systematically review the traditional uses, phytochemistry, pharmacodynamics, pharmacokinetics and toxicity of FTB. The crude herb FTB contains more than 100 chemical constituents in 11 categories with 13 possible pharmacological activities. Included studies revealed that these pharmacological activities may be mainly attributed to the alkaloids isolated from FTB. Its multiple compounds and effects may contribute to novel drug discovery for the management of multiple conditions. Current studies indicated that additional or even synergistic therapeutic effects could be produced, and may be superior to a single component, when combined multiple active constituents are used, because of the concurrent and selective interactions with multiple target proteins of a disease or a condition, such as malignant tumor, respiratory disorders and inflammation. Classic literature also suggested that there are multiple actions which may be performed by FTB. However, the mechanisms of actions of FTB are still not clear. The relationship between active chemical constituents and possible mechanisms needed to be systematically evaluated as well. High quality and well-designed in vivo and in vitro studies on the mechanisms of actions, pharmacokinetic and toxicity of FTB are recommended. A comprehensive and thorough investigation of the multi-target network pharmacology of FTB could provide insights into various conditions, and thus support evidence-based practice.

## Figures and Tables

**Table 1:** Summary of the 134 chemical constituents isolated from Fritillariae Thunbergii Bulbus.

No.	Derivatives and Constitutions	Molecular Formula	PubChem CID/SID	Molecule Weight (g/mol)	Method	
					SRV Group	SNRV Group
**A**	**Alkaloids (*n* = 26)**					
A1	Peimine	C_27_H_45_NO_3_	131900	431.661	HPLC [33]; HPLC-ELSD [44,60,63]; HPLC-ESI/MS [70]; HPLC-MS [21]; UPLC-ELSD [26]; LC/ESI-QTOF-MS/MS [72]; Pre-column derivatization HPLC [38]; GC [55]; ^1^HNMR/^13^CNMR/HR-FAB-MS [8]	HPLC [18,20]; HPLC-ELSD [17,27,28,29,32,36,47,51]; HSCCC-ELSD [31]; UPLC-CAD/HPLC-ELSD [42]; UHPLC-MS/MS [59]; LC-LTQ-Orbitrap MS^n^ [22]; LC-TRAP-MS/LC-ESI-MS [45]; ESI-MS [73]; TLCS [23,48,57]; ^13^CNMR [69]; Determination of colorimetry [23]; RRLC-MS/MS [49]; MS/IR/^1^HNMR/^13^CNMR [75]; GC [37]; GC direct [17]; Indirect UV detection [41]; Paper partition chromatography [46]; Aid-dye ofnrimetry [24,39]
A2	Peiminine	C_27_H_43_NO_3_	167691	429.645	HPLC-MS [21]; HPLC-ELSD [60]; HPLC-ESI/MS [70]; Pre-column derivatization HPLC [38]; UPLC-ELSD [26]; LC/ESI-QTOF-MS/MS [72]; GC [56]; ^1^HNMR/^13^CNMR/HR-FAB-MS [8]; Acid dye two-phase titration [50]	HPLC [17,18,20]; HPLC-ELSD [27,28,29,32,36,47]; HPLC-CAD/HPLC-ELSD [42]; HSCCC-ELSD [31]; ESI-MS [73]; UHPLC–MS/MS [59]; TLCS [23,48,57]; LC-LTQ-Orbitrap MS^n^ [22]; LC-TRAP-MS/LC-ESI-MS [45]; ^13^CNMR [69]; Determination of colorimetry [23]; RRLC-MS/MS [49]; MS/IR/^1^HNMR/^13^CNMR [75]; GC [37]; GC direct [17]; Indirect UV detection [41]
A3	Zhebeinine	C_27_H_45_NO_3_	21121503	431.661	N/A	TLC [69]; ^13^CNMR [69]
A4	Zhebeinone	C_27_H_43_NO_3_	NF (Figure 2)	429.645	N/A	GC-MS [65]
A5	Ebeiedine	C_27_H_45_NO_2_	101324888	415.662	HPLC [33]; GC [56]; LC/ESI-QTOF-MS/MS [72]; ^1^HNMR/^13^CNMR/HR-FAB-MS [8]	GC [37]; GC direct [17]; LC-TRAP-MS/LC-ESI-MS [45]
A6	Ebeiedinone	C_27_H_43_NO_2_	102062796	413.646	GC [56]; ^1^HNMR/^13^CNMR/HR-FAB-MS [8]	GC [37]; GC direct [17]; LC-TRAP-MS/LC-ESI-MS [44]; LC-LTQ-Orbitrap MS^n^ [22]; HPLC-ELSD [17,36]
A7	Isoverticine	C_27_H_45_NO_3_	21573744	431.661	GC [56]; LC/ESI-QTOF-MS/MS [72]; ^1^HNMR/^13^CNMR/HR-FAB-MS [8]	GC [37]; GC direct [17]; LC-TRAP-MS/LC-ESI-MS [45]; HPLC-ELSD [17,36]; ESI-MS [73]; TLCS [57]
A8	Suchengbeisine	C_27_H_43_NO_3_	102112537	429.645	HPLC [33]; ^1^HNMR/^13^CNMR/HR-FAB-MS [8]	N/A
A9	Peimisine	C_27_H_41_NO_3_	161294	427.629	LC/ESI-QTOF-MS/MS [72]; UPLC-ELSD [26]	LC-TRAP-MS/LC-ESI-MS [45]; HPLC-ELSD [17,36,47]; TLC [68]; TLCS [57]; LC-LTQ-Orbitrap MS^n^ [22]; RRLC-MS/MS [49]
A10	Peimisine-N-oxide	C_27_H_42_NO_4_	NF	444.636	N/A	LC-LTQ-Orbitrap MS^n^ [22]
A11	Zhebeininoside	C_33_H_55_NO_8_	NF (Figure 2)	593.802	LC/ESI-QTOF-MS/MS [72]	LC-TRAP-MS/LC-ESI-MS [45]; LC-LTQ-Orbitrap MS^n^ [22]; ^13^CNMR/Infrared spectra analysis [67]; ESI-MS [73]
A12	Verticinone-3-β-d-glucoside	C_33_H_53_NO_8_	90479257	591.786	LC/ESI-QTOF-MS/MS [72]	N/A
A13	Puqietinone	C_28_H_47_NO_2_	10693900	429.689	LC/ESI-QTOF-MS/MS [72]	N/A
A14	Puqiedinone	C_27_H_43_NO_2_	126149	413.646	LC/ESI-QTOF-MS/MS [72]	LC-TRAP-MS/LC-ESI-MS [45]; LC-LTQ-Orbitrap MS^n^ [22]
A15	Puqiedine	C_27_H_45_NO_2_	101400593	415.662	LC/ESI-QTOF-MS/MS [72]	LC-TRAP-MS/LC-ESI-MS [45]; ESI-MS [73]
A16	Eduardine	C_27_H_43_NO_2_	NF (Figure 2)	413.646	N/A	GC-MS [66]
A17	Zhebeirine	C_27_H_43_NO_2_	NF (Figure 2)	413.646	N/A	GC-MS [66]; ESI-MS [73]
A18	2,3-deoxyladenosine	C_10_H_11_N_5_O_2_	NF	233.231	N/A	ESI-MS [73]
A19	N-demethylpuqietinone	C_27_H_45_NO_2_	11304576	415.662	N/A	ESI-MS [73]
A20	Puqienine B	C_28_H_45_NO_2_	11419389	443.672	N/A	ESI-MS [73]
A21	Zhebeinone-3-β-d-glucoside	C_33_H_55_NO_8_	NF (Figure 2)	593.802	N/A	ESI-MS [73]
A22	Peiminoside	C_33_H_55_NO_7_	90479565	577.803	N/A	Paper partition chromatography [46]
A23	Frithunbol A	C_27_H_41_NO_4_	NF (Figure 2)	443.628	^1^HNMR/^13^CNMR/HR-FAB-MS [8]	N/A
A24	Frithunbol B	C_27_H_42_NO_3_	NF (Figure 2)	428.637	^1^HNMR/^13^CNMR/HR-FAB-MS [8]	N/A
A25	Eduardinine	C_27_H_45_NO_2_	NF (Figure 2)	415.662	^1^HNMR/^13^CNMR/HR-FAB-MS [8]	N/A
A26	3β-hydroxy-5α-jervanin-12-en-6-one	C_27_H_42_NO_3_	NF (Figure 2)	428.637	^1^HNMR/^13^CNMR/HR-FAB-MS [8]	N/A
**B**	**Compounds from essential oils (*n* = 29)**					
B1	δ-elemene	C_15_H_24_	12309449	204.357	N/A	GC-MS [16]
B2	δ-selinene	C_15_H_24_	520383	204.357	N/A	GC-MS [16]
B3	Tetradecanoic acid	C_14_H_28_O_2_	11005	228.376	N/A	GC-MS [16]
B4	Pentadecanoic acid	C_15_H_30_O_2_	13849	242.403	N/A	GC-MS [16]
B5	Hexadecanoic acid, methyl ester	C_17_H_34_O_2_	8181	270.457	N/A	GC-MS [16,25]
B6	9-hexadecenoic acid	C_16_H_30_O_2_	5282745	254.414	N/A	GC-MS [16]
B7	N-hexadecanoic acid	C_16_H_32_O_2_	985	256.43	N/A	GC-MS [16]
B8	Kaur-15-ene	C_20_H_32_	521318	272.476	N/A	GC-MS [16]
B9	Heptadecanoic acid	C_17_H_34_O_2_	10465	270.457	N/A	GC-MS [16]
B10	Kaurene	C_20_H_32_	91746569	272.476	N/A	GC-MS [16]
B11	9,12-Octadecadienoic acid (Z,Z)-, methyl ester	C_19_H_34_O_2_	5284421	294.479	N/A	GC-MS [16]
B12	9-Tetradecenal, (Z)-	C_14_H_26_O	5364471	210.361	N/A	GC-MS [16]
B13	9,12-Octadecadienoic acid, methyl ester, (E, E)-	C_19_H_34_O_2_	135058711	294.479	N/A	GC-MS [16]
B14	Oleic acid	C_18_H_34_O_2_	445639	282.468	N/A	GC-MS [16]
B15	Linoleic acid, ethyl ester	C_2036_O_2_	5282184	308.506	N/A	GC-MS [16]
B16	Butylated hydroxytoluene	C_15_H_24_O	31404	220.356	N/A	GC-MS (Du et al., 2018)
B17	L-(+)-Ascorbic acid 2,6- dihexadecanoate	C_38_H_68_O_8_	54722209	652.954	N/A	GC-MS (Du et al., 2018)
B18	Ethyl 9-hexadecenoate	C_18_H_34_O_2_	5364759	282.468	N/A	GC-MS [25]
B19	Hexadecanoic acid, ethyl ester	C_18_H_36_O_2_	12366	284.484	N/A	GC-MS [25]
B20	1H-Naphtho [2,1-B] pyran, 3-ethenyldodecahydro-3,4a,7,7,10a-pentamethyl-	C_20_H_34_O	273540178	290.491	N/A	GC-MS [25]
B21	Kaur-16-ene	C_20_H_32_O	520687	272.476	N/A	GC-MS [25]
B22	9,11-Octadecadienoic acid, methyl ester, (E, E)-	C_19_H_34_O_2_	319301067	294.479	N/A	GC-MS [25]
B23	9,12-Octadecadienoic acid	C_18_H_32_O_2_	5282457	280.452	N/A	GC-MS [25]
B24	Linoleic acid, ethyl ester	C_20_H_36_O_2_	5282184	308.506	N/A	GC-MS [25]
B25	Octadecanoic acid, ethyl ester	C_20_H_40_O_2_	8122	312.538	N/A	GC-MS [25]
B26	2(1H)-Phenanthrenone, 3,4,4a,4b,5,6,7,8,10,10a-decahydro- 1,1,4a,7,7-pentamethyl, [4aR-(4a.α., 4b.β.,10a.β.)]-	C_19_H_30_O	621255	274.448	N/A	GC-MS [25]
B27	3-Methyleneandrostan-17-ol	C_20_H_32_O	625647	288.475	N/A	GC-MS [25]
B28	Androst-4-en-3-one, 17-hydroxy-, (17.β.)	C_19_H_28_O_2_	50049744	288.431	N/A	GC-MS [25]
B29	Podocarp-7-en-3.β.-ol, 13.β.-methyl-13-vinyl-	C_20_H_32_O	620519	288.475	N/A	GC-MS [25]
**C**	**Diterpenoids (*n* = 13)**					
C1	Isopimaran-19-ol	C_20_H_32_O	75399514	288.475	N/A	^1^HNMR/^13^CNMR [35]
C2	Isopimaran-19-oic acid, methyl ester	C_21_H_32_O_2_	NF (Figure 2)	316.485	N/A	^1^HNMR/^13^CNMR [34,35]
C3	Ent-kauran-16β, 17-diol	C_20_H_34_O_2_	NF (Figure 2)	306.49	N/A	TLC [68]; ^1^HNMR/^13^CNMR [34,35]
C4	Ent-kauran-16α, 17-diol	C_20_H_34_O_2_	NF (Figure 2)	306.49	N/A	^1^HNMR/^13^CNMR [34,35]
C5	Ent-16β, 17-epoxy-kaurane	C_20_H_32_O	79592848	288.475	N/A	^1^HNMR/^13^CNMR [35]
C6	Ent-16α-methoxy-kauran-17-ol	C_21_H_36_O_2_	51842049	320.517	N/A	^1^HNMR/^13^CNMR [35]
C7	Ent-kaur-15-en-17-ol	C_20_H_32_O	3082069	288.475	N/A	^1^HNMR/^13^CNMR [35]
C8	Trans-communol	C_20_H_32_O	51909318	288.475	N/A	^1^HNMR/^13^CNMR [34]
C9	Trans-comminic acid, methyl ester	C_21_H_32_O_2_	NF (Figure 2)	316.485	N/A	^1^HNMR/^13^CNMR [34]
C10	Ent-17-norkauran-16-one	C_19_H_30_O	12740861	274.448	N/A	^1^HNMR/^13^CNMR [34]
C11	Ent-15β,16-epoxy-kauran-17-ol	C_20_H_32_O_2_	51511087	304.474	N/A	^1^HNMR/^13^CNMR [34]
C12	Ent-16β-hydroxy-kauran-17-yl ent-kaur-15-en-17-oate	C_40_H_63_O_3_	NF (Figure 2)	591.941	N/A	^1^HNMR/^13^CNMR [34]
C13	Ent-(16S)-atisan-13, 17-oxide	C_20_H_32_O	50418337	288.475	N/A	^1^HNMR/^13^CNMR [34]
**D**	**Carbohydrates (*n* = 2)**					
D1	β-d-glucose4-1β-d-galactose	C_12_H_22_O_11_	NF	342.297	N/A	HPLC-ELSD [19]
D2	Sucrose	C_12_H_22_O_11_	5988	342.297	N/A	HPLC-ELSD [19]
**E**	**Sterols (*n* = 2)**					
E1	β-sitosterol	C_29_H_50_O	222284	414.718	N/A	TLC [68]
E2	Daucosterol	C_35_H_60_O_6_	5742590	576.859	N/A	TLC [68]
**F**	**Amino acids (*n* = 18)**					
F1	Glycine	C_2_H_5_NO_2_	750	75.067	N/A	HPLC [64]
F2	Leucine	C_6_H_13_NO_2_	6106	131.175	N/A	HPLC [64]
F3	Methionine	C_5_H_11_NO_2_S	6137	149.208	N/A	HPLC [64]
F4	Tyrosine	C_9_H_11_NO_3_	6057	181.191	N/A	HPLC [64]
F5	Histidine	C_6_H_9_N_3_O_2_	6274	155.157	N/A	HPLC [64]
F6	Threonine	C_4_H_9_NO_3_	6288	119.12	N/A	HPLC [64]
F7	Alanine	C_3_H_7_NO_2_	5950	89.094	N/A	HPLC [64]
F8	Isoleucine	C_6_H_13_NO_2_	6306	131.175	N/A	HPLC [64]
F9	Tryptophan	C_11_H_12_N_2_O_2_	6305	204.229	N/A	HPLC [64]
F10	Cystine	C_6_H_12_N_2_O_4_S_2_	67678	240.292	N/A	HPLC [64]
F11	Lysine	C_6_H_14_N_2_O_2_	5962	146.19	N/A	HPLC [64]
F12	Aspartic acid	C_4_H_7_NO_4_	5960	133.103	N/A	HPLC [64]
F13	Valine	C_5_H_11_NO_2_	6287	117.148	N/A	HPLC [64]
F14	Phenylalanine	C_8_H_8_O_2_	6140	165.192	N/A	HPLC [64]
F15	Proline	C_5_H_9_NO_2_	145742	115.132	N/A	HPLC [64]
F16	Serine	C_3_H_7_NO_3_	5951	105.093	N/A	HPLC [64]
F17	Glutamic acid	C_5_H_9_NO_4_	33032	147.13	N/A	HPLC [64]
F18	Arginine	C_6_H_14_N_4_O_2_	6322	174.204	N/A	HPLC [64]
**G**	**Nucleosides (*n* = 6)**					
G1	Uridine	C_9_H_12_N_2_O_6_	6029	244.203	LC-ESI-MS^n^ [70]; HPLC-UV-ESI/MS [70]; HPLC [71]	HPLC [64]
G2	Guanosine	C_10_H_13_N_5_O_5_	6802	283.244	LC-ESI-MS^n^ (Zhang, 2008); HPLC-UV-ESI/MS [70]; HPLC [71]	HPLC [64]
G3	Adenosine	C_10_H_13_N_5_O_4_	60961	267.245	LC-ESI-MS^n^ (Zhang, 2008); HPLC-UV-ESI/MS [70]; HPLC [71]	HPLC [64]
G4	Thymidine	C_10_H_14_N_2_O_5_	5789	242.231	LC-ESI-MS^n^ [70]; HPLC-UV-ESI/MS [70]	HPLC [64]
G5	Cytidine	C_9_H_13_N_3_O_5_	6175	243.219	N/A	HPLC [64]
G6	Inosine	C_10_H_12_N_4_O_5_	6021	268.229	N/A	HPLC [64]
**H**	**Nucleobases (*n* = 4)**					
H1	Adenine	C_5_H_5_N_5_	190	135.13	LC-ESI-MS^n^ (Zhang, 2008); HPLC-UV-ESI/MS (Zhang, 2008)	HPLC [64]
H2	Hypoxanthine	C_5_H_4_N_4_O	790	136.114	N/A	HPLC [64]
H3	Uracil	C_4_H_4_N_2_O_2_	1174	112.088	N/A	HPLC [64]
H4	Thymine	C_5_H_6_N_2_O_2_	1135	126.115	N/A	HPLC [64]
**I**	**Fatty acids (*n* = 4)**					
I1	Vernolic acid	C_18_H_32_O_3_	6449780	296.451	N/A	ESI-MS [73]
I2	2-monopalmitin	C_19_H_38_O_4_	123409	330.509	N/A	ESI-MS [73]
I3	13(R)-hydroxy-octadeca-(9Z,11E ,15Z)-trien-oic acid	C_18_H_30_O_3_	643726	294.435	N/A	ESI-MS [73]
I4	Picropodophyllotoxin	C_22_H_22_O_8_	72435	414.41	N/A	^13^CNMR/Infrared spectra analysis [67]
**J**	**Lignans (*n* = 3)**					
J1	Octahydrocurcumin	C_21_H_28_O_6_	11068834	376.449	N/A	ESI-MS [73]
J2	Zhebeiresinol	C_14_H_16_O_6_	192547	280.276	N/A	ESI-MS [73]; ^1^HNMR/^13^CNMR [30]
J3	Sauriol B	C_21_H_28_O_6_	15965508	376.449	N/A	ESI-MS [73]
**K**	**Elements (*n* = 27)**					
K1	Aluminum	Al	5359268	26.982	ICP-OES [62]	FAAS [61]; GFAAS [61]
K2	Arsenic	As	5359596	74.922	ICP-AES [43]; ICP-OES [14]	ICP-AES [74]; ICP-OES [15]; ICAP [55]; FAAS [61]; GFAAS [61]
K3	Boron	B	5462311	10.81	ICP-OES [14,62]	N/A
K4	Barium	Ba	5355457	137.327	ICP-AES [58]; ICP-OES [14]	N/A
K5	Bismuth	Bi	5359367	208.98	ICP-OES [14]	N/A
K6	Calcium	Ca	5460341	40.078	ICP-OES [62]	FAAS [52,53,61]; GFAAS [61]
K7	Cadmium	Cd	23973	112.414	ICP-AES [43]; ICP-OES [14,62]	ICP-AES [74]; ICP-OES [15]; ICAP [55]; FAAS [61]; GFAAS [61]; AAS [40]
K8	Cobalt	Co	104730	58.933	ICP-AES [58]; ICP-OES [14]	FAAS [61]; GFAAS [61]
K9	Chromium	Cr	23976	51.996	ICP-AES [58]	FAAS [61]; GFAAS [61]; AAS [40]
K10	Copper	Cu	23978	63.546	ICP-AES [43,58]; ICP-OES [14]	ICP-AES [74]; ICP-OES [15]; ICAP [55]; FAAS [52,53,61]; GFAAS [61]; AAS [40]
K11	Iron	Fe	23925	55.845	ICP-AES [43,58]; ICP-OES [14]	ICP-AES [74]; FAAS [52,53,61]; GFAAS [61]; AAS [40]
K12	Mercury	Hg	23931	200.592	ICP-AES [43]; ICP-OES [14]	ICP-OES [15]
K13	Indium	In	5359967	114.818	ICP-OES [14]	N/A
K14	Potassium	K	5462222	39.098	ICP-OES [62]	FAAS [53,61]; GFAAS [61]
K15	Lithium	Li	3028194	6.94	ICP-AES [43]	N/A
K16	Magnesium	Mg	5462224	24.305	ICP-AES [43]; ICP-OES [62]	FAAS [53,61]; GFAAS [61]; AAS [40]
K17	Manganese	Mn	23930	54.938	ICP-AES [43,58]; ICP-OES [14]	ICP-AES [74]; FAAS [53,61]; GFAAS [61]; ICAP [55]
K18	Molybdenum	Mo	23932	95.95	ICP-OES [62]	N/A
K19	Sodium	Na	5360545	22.99	ICP-OES [62]	FAAS [53,61]; GFAAS [61]
K20	Nickel	Ni	935	58.693	ICP-AES [58]; ICP-OES [14,62]	ICAP [55]
K21	Phosphorus	P	5462309	30.974	ICP-OES [62]	N/A
K22	Lead	Pb	5352425	207.2	ICP-AES [43]; ICP-OES [14]	ICP-OES [15]; AAS [40]; ICAP [55]
K23	Sulfur	S	5362487	32.06	ICP-AES [43]	N/A
K24	Selenium	Se	6326970	78.971	N/A	Spectrophotometry [54]
K25	Strontium	Sr	5359327	87.62	ICP-AES [43,58]; ICP-OES	FAAS [61]; GFAAS [61]
K26	Vanadium	V	23990	50.941	ICP-AES [58]	N/A
K27	Zinc	Zn	23994	65.379	ICP-AES [43,58]; ICP-OES [14]	ICP-AES [74]; ICP-OES; ICAP [55]; FAAS [52,53,61]; GFAAS [61]; AAS [40]

Note: N/A: Not applicable; NF: Not found; SRV: Studies reporting a voucher number; SNRV: Studies not reporting voucher number; Corresponding molecular structures refer to PubChem and Figure 2.

**Table 2:** Characteristics of the 23 included studies relevant to the mechanisms of actions of Fritillariae Thunbergii Bulbus.

Pharmacological Effects/ Included Studies	Study Type	Extract	Characteristics of the Sample	Interventions	Duration	Primary Results
**1. Anti-cancer**						
Li et al., 2013 [80]	In vitro	Total alkaloids	Human lung adenocarcinoma parental cells A549; Resistant cells A549/DDP	Cytotoxicity: FTB 12.5, 25, 50, 100, 200 mg/L and vehicle 0.5% CMC-Na. Multidrug resistance reversal effect: FTB 9 mg/L, cyclosporine A 1 mg/L or tetrandrine 1 mg/L plus DDP (final concentration: 0.01, 0.1, 1, 10, 100 mg/L). MDR1 mRNA and P-gp expression: A549/DDP + vehicle; A549/DDP + FTB 9 mg/L; A549/DDP + DDP 14 mg/L; A549/DDP + DDP 14 mg/L + FTB 9 mg/L; A549 + vehicle	72 h	IC_50_ of TAF to A549: 141 ± 5 mg/L; IC_50_ of TAF to A549/DDP: 298 ± 22 mg/L; FTB was superior to closporine A and tetrandrines in increasing the reversal fold; FTB alone was superior to vehicle in decreasing the MDR1 mRNA and P-gp expression.
	In vivo	Total alkaloids	60 BALB/c nude mice (A549/DDP model)	Vehicle 0.5% CMC-Na; DDP 5 mg/kg, ig, qd; TAF 2 mg/kg, ig, qd; DDP 5 mg/kg + FTB 0.5 mg/kg, ig, qd; DDP 5 mg/kg + FTB 1 mg/kg, ig, qd; DDP 5 mg/kg + FTB 2 mg/kg, ig, qd	13 days	DDP + TAF was superior to DDP alone in increasing the tumor inhibitory rate.
Liu et al., 2015 [9]	In vitro	Total alkaloids; Total nucleosides	Human hepatocellular carcinoma cell line HepG2, Resistant cell line HepG2/MDR	Blank control; P-gp positive inhibitor verapamil; Total alkaloid, nucleoside, or polysaccharide 5 μg/mL respectively; Total alkaloid, nucleoside, or polysaccharide 50 μg/mL respectively	1 h	Total alkaloids or Total nucleosides in FTB was superior to the control in increasing the restriction of efflux activity of P-gp.
Yang et al., 2005 [91]	In vitro	Aqueous extract	Human lung adenocarcinoma parental cells LM_2_	Blank control; FZ + FTB (75 + 75, 25 + 25, 5 + 5, 1 + 1 mg/mL respectively); FZ (75, 25, 5 mg/mL respectively); FTB (75, 25, 5 mg/mL respectively)	48 h	FTB alone was superior to control and FZ + FTB in increasing the apoptosis rate.
	In vivo	Aqueous extract	142 SPF C_57_ mice (Human lung adenocarcinoma parental cells LM_2_ model)	Blank control; FZ + FTB (0.8 + 1.6 g/kg, 0.64 + 1.28 g/kg, 0.51 + 1.02 g/kg respectively, ig, qd); FZ (0.8 g/kg, 0.64 g/kg, 0.51 g/kg respectively, ig, qd); FTB (1.6 g/kg, 1.28 g/kg, 1.02 g/kg respectively, ig, qd)	18 days	FTB alone was superior to control and FZ + FTB in increasing the tumor inhibitory rate and reducing the number of metastases.
Tong, 2016 [49]	In vitro	Peimine; Peiminine	Human breast carcinoma cells MCF-7; Resistant cell line MCF-7/ADM	Peimine 12.5–400 μg/mL; Peiminine 12.5–400 μg/mL; ADM (0.78125–100 μg/mL) + Peimine or Peiminine; Paclitaxel (2.5–80 μg/mL) + Peimine or Peiminine	48 h	Both peimine and peiminine could reverse the multi-drug resistant tumor resistance of ADM or paclitaxel
**2. Tracheobronchial relaxation**						
Chan, 2000 [17]	In vitro	Peimine; Peiminine; Ebeiedine	Rat tracheal and bronchial rings	Peimine, Peiminine, Ebeiedine, Imperialine, puqietinone, Salbutamol, Diphenhydramine, Codeine cumulative concentrations 1 nM-100 μM respectively	Immediate	Peimine, peiminine, ebeiedine and puqietinone in FTB could relax the tracheobronch of rats.
Wu et al., 2018 [56]	In vitro	Total alkaloids	Rat tracheal and bronchial rings	Total alkaloids cumulative concentrations 0-3 g/mL	Immediate	Total alkaloids in FTB were superior to control in increasing the pD_2_ value.
**3. Antitussive**						
Yan et al., 2012 [90]	In vivo	Micro powders	44 guinea pigs (citric acid induced cough model)	Blank control: purified water, ig,qd; AZTB: 1.5 g/kg, ig, qd; FTB: 1.5 g/kg, ig, qd; Codeine phosphate: 0.02 g/kg, ig, qd	5 min	FTB was superior to control in reduding the frequency of cough and prolonging the remission period.
Yan et al., 2012 [89]	In vivo	Micro powders	53 guinea pigs (citric acid induced cough model)	Blank control: purified water, ig,qd; FTB: 1.5 g/kg, ig, qd; AZTB: 1.5 g/kg, ig, qd; WBBM: 1.5 g/kg, ig, qd; Codeine phosphate: 0.02 g/kg, ig, qd	5 min	FTB was superior to control in reduding the frequency of cough and prolonging the remission period.
Guo, 2007 [27]	In vivo	Aqueous extract	140 Kunming mice (ammonium hydroxide induced cough model)	Blank control: NS, ig,qd; FTB: 0.026, 0.052, 0.104 g/kg respectively, ig, qd; Codeine phosphate: 0.03 g/kg, ig, qd	3 days	FTB harvested in all four places was superior to control in reduding the frequency of cough and prolonging the remission period.
**4. Expectorant**						
Yan et al., 2012 [89]	In vivo	Fine powders	40 mice	Blank control: purified water, ig,qd; FTB: 2 g/kg, ig, qd; AZTB: 2 g/kg, ig, qd; WBBM: 2 g/kg, ig, qd; Ammonium chloride: 1 g/kg, ig, qd	5 days	Fine powders of FTB was superior to control in reducing the amount of phlegm secretion.
Wang et al., 1993 [86]	In vivo	Alcohol extract	40 Wistar rats	Control: starch paste, 15 g/kg, ig,qd; FTB: 15 g/kg, ig, qd; CBM: 15 g/kg, ig, qd; WBM: 15 g/kg, ig, qd	5 h	Alcohol extract of FTB was superior to control in reducing the amount of phlegm secretion.
**5. Anti-inflammation**						
Kim et al., 2016 [33]	In vitro	Peimine; Ebeiedine; Suchengbeisine	Human mucoepidermoid carcinoma cells NCI-H292	Peimine; Ebeiedine; Suchengbeisine	24 h	Peimine, ebeiedine, or suchengbeisine was superior to control in decreasing the expression of MUC5AC mucin gene.
Zhou et al., 2017 [73]	In vitro	Puqiedine; Zhebeiresinol, 2-monopalmitin, N-demethylpuqietinone; Isoverticine	Human embryonic kidney cells HEK293	FTB 10, 3, 1 mg/mL respectively; Dexamethasone 10^−5^ mol/L	6 h	Puqiedine, zhebeiresinol, 2-monopalmitin, n-demethylpuqietinone or isoverticine was superior to control in reducing the expression of NF-Κb level in cells.
Xia et al., 2011 [87]	In vivo	Aqueous extract	30 mice (CP/CPPS model)	Blank control and blank normal: NS; FTB 0.1 mL/10 g, ig, qd	7 days	Aqueous extract of FTB was superior to control in relieving the inflammation and over proliferation of fibroblasts of the prostate and reducing the serum level of nitric oxide in mice.
Zhang et al., 1998 [93]	In vivo	Alcohol extract	40 ICR mice (xylene induced ear swelling)	Blank control: Purified water, ig,qd; Ethenzamide 0.3 g/kg, ig,qd; FTB 0.8, 2.4 g/kg respectively, ig, qd	4 h	Alcohol extract of FTB was superior to control in releving the swelling of ear.
	In vivo	Alcohol extract	40 ICR mice (carrageenin induced foot plantar swelling)	Blank control: Purified water, ig,qd; Ethenzamide 0.3 g/kg, ig,qd; FTB 0.8, 2.4 g/kg respectively, ig, qd	6 h	Alcohol extract of FTB was superior to control in releving the swelling of foot plantar.
Xu et al., 2016 [88]	In vitro	Peimine	Human Embryonic Kidney Cells HEK293	Peimine concentrations 1, 3 10, 30, 100, 300 μM	300 s	Peimine could inhibit the Nav 1.3 channel.
**6. Pain suppression**						
Zhang et al., 1998 [92]	In vivo	Alcohol extract	44 ICR mice (acetic acid induced pain)	Blank control: Purified water, ig,qd; Ethenzamide 0.3 g/kg, ig,qd; FTB 0.8, 2.4 g/kg respectively, ig, qd	8 h	Alcohol extract of FTB was superior to control in reducing the frequency of mouse writhing.
			40 ICR mice (thermal stimulus pain)	Blank control: Purified water, ig,qd; Ethenzamide 0.3 g/kg, ig,qd; FTB 0.8, 2.4 g/kg respectively, ig, qd	3 h	Alcohol extract of FTB was superior to control in increasing the remission period of tail-flick latency.
Xu et al., 2016 [88]	In vitro	Peimine	Human Embryonic Kidney Cells HEK293	Peimine concentrations 1, 3 10, 30, 100, 300 μM	300 s	Peimine could inhibit the Nav 1.7 channel.
**7. Antioxidation**						
Ruan et al., 2016 [85]	In vitro	Total alkaloids	DPPH radical; ABTS radical; FRAP reagent	DPPH: Total alkaloids 50 μL; ABTS: Total alkaloids 50 μL; FRAP: Total alkaloids 20 μL, control ethanol 20 μL	DPPH: 30 min; ABTS: Immediate; FRAP: Immediate	Total alkaloids in FTB have strong a antioxidative capacity evidence by the results of DPPH, ABTS and FRAP.
Ma, 2014 [84]	In vitro	polysaccharide	DPPH radical	polysaccharide 0.1, 0.2, 0.4, 0.6, 0.8, 1 mg/mL	30 min	Polysaccharide in FTB has strong antioxidative capacity evidence by the results of DPPH and absorption spectroscopy test.
**8. Antiulcer**						
Zhang et al., 2018 [92]	In vivo	Alcohol extract	90 SD rats (oral ulcer model)	Blank model: NS; FTB 4, 2, 1 g/kg respectively, external, qid; Gui Lin Xi Gua Shuang, 1 g/kg, external, qid	6 days	Alcohol extract of FTB was superior to control in inhibiting oral ulcer.
	In vivo	Alcohol extract	120 ICR mice (gastric ulcer model)	Blank control: Purified water, ig,qd; Mepirizole 0.05 g/kg, ig,qd; FTB 0.8, 2.4 g/kg respectively, ig, qd;	2 h	Alcohol extract of FTB was superior to control in inhibiting gastric ulcer.
**9. Anti-muscarinic**						
Zhou et al., 2006 [75]	In vitro	Peimine; Peiminine; Puqietinone	Human Embryonic Kidney Cells HEK293	Five alkaloids: 10μM (final concentration each); Negative control: carbachol, 0.3 μM (final concentration each); Positive control: Atropine 1 μM (final concentration each)	48 h	Peimine, peiminine or puqietinone was superior to control in raising the cAMP level in cells transfected with muscarinic M_2_ receptor.
**10. Anti-thyroid**						
Lin et al., 2010 [82]	In vivo	Extract	180 SD rats; 60 SPF mice (hyperthyroidism model)	Blank control and blank model: NS, ig, qd; Tapazole 0.02 g/kg, ig, qd; FTB 3, 1.5, 0.75 g/kg respectively, ig, qd	14 days	FTB was superior to control in reducing the serum level of T_3_, T_4_, cAMP, cGMP and raising the abilities of hypoxia tolerance.
**11. Regulation of blood rheology**						
Jiang et al., 2002 [78]	In vivo	Aqueous extract	50 SD rats	Blank control: Purified water, ig,qd; FTB 1 mL respectively, ig, qd	1 week	Aqueous extract of FTB was superior to control in reducing the whole blood viscosity, restricting the trythrocyte aggregation and raising the erythrocyte deformability.
**12. Anti-diarrhea**						
Zhang et al., 1998 [93]	In vivo	Alcohol extract	40 ICR mice (castor oil induced diarrhea); 40 ICR mice (FXY induced diarrhea)	Blank control: Purified water, ig,qd; Ethenzamide 0.3 g/kg, ig,qd; FTB 0.8, 2.4 g/kg respectively, ig, qd	8 h	Alcohol extract of FTB was superior to control in reducing the frequency of diarrhea.
**13. Neuroprotection**						
Suh et al., 2018 [8]	In vitro	Frithunbol B; Ebeiedinone; 3β-hydroxy-5α-jervanin-12-en-6-one; Suchengbeisine	lipopolysaccharide-activated BV-2 cells; C6 glioma cells	NO study: 100 ng/mL isolates; NGF study: 20 μM isolates	24 h	Frithunbol B, ebeiedinone, and suchengbeisine significantly reduce the nitric oxide level, compared to control; 3β-hydroxy-5α-jervanin-12-en-6 -one was superior to control in increasing the nerve growth factor level.

Note: ABTS: 2,2′-azino-bis (3-ethylbenzothiazoline-6-sulphonic acid); AZTB: An zi bei mu, Fritillaria unibracteata Hsiao et K. C. Hsia; CBM: Chuan bei mu, Fritillariae Cirrhosae Bulbus; CMC-Na: Sodium carboxyl methyl cellulose; CP/CPPS: Chronic prostatitis/chronic pelvic pain syndrome; DDP: Cisplatin; DPPH: 2,2-diphenyl-1-picrylhydrazyl; FRAP: Ferric reducing capacity; FTB: Fritillariae Thunbergii Bulbus, Zhe bei mu; FXY: Fan xie ye, Sennae Folium; FZ: Fu zi, Aconiti Radix Lateralis Praeparata; ICR: Institute of Cancer Research; NF-κB: Nuclear factor-κB; NGF: Nerve growth factor; NO: Nitric oxide; NS: Normal saline; P-gp: P-glycoprotein; SD: Sprague Dawley; SPF: Specific-pathogen-free; WBM: Wan bei mu, Fritillaria anhuiensis S.C.Chen et S.P.Yin; WBBM: Wa bu bei mu, Fritillaria unibracteata Hsiaoet K. C. Hsia var. wabuensis (S. Y. Tang et S. C. Yue) Z. D. Liu, S. Wang et S.C. Chen.

**Table 3:** Pharmacokinetics of peimine (A1) and peiminine (A2) and peimisine (A9) in Fritillariae Thunbergii Bulbus.

Included Studies	Study Type	Methods	Animals	Interventions	T_1/2_ (h)	T_max_ (h)	CL/F (L/h/kg)	V/F (L/kg)	C_max_ (μg/L)	AUC_0-t_ (μg h/L)	AUC_0-__∞_ (μg h/L)
Chen et al., 2011 [76]	In vivo	LC-MS-MS; DAS 2.0 package	12 female SD rats	Peimine, 4.25 g/kg, ig	4.8 ± 0.8	1.5 ± 0.6	119.6 ± 40.1	854.8 ± 363.9	43.2 ± 5.4	N/A	260.5 ± 119.8
				Peiminine, 4.25 g/kg, ig	6.6 ± 3.2	4.5 ± 1.9	34.1 ± 4.8	321.1 ± 155.4	57.6 ± 23.0	N/A	618.3 ± 94.8
Chen et al., 2013 [77]	In vivo	LC-MS-MS; DAS 2.0 package	6 female SD rats	Peimine, 4.25 g/kg, ig	4.2 ± 2.0	1.5 ± 0.7	128.9 ± 32.6	781.3 ± 305.6	43.7 ± 22.7	214.2 ± 84.6	214.3 ± 84.5
				Peiminine, 4.25 g/kg, ig	3.4 ± 1.7	2.8 ± 0.9	36.3 ± 15.8	268.8 ± 163.9	64.2 ± 40.0	571.0 ± 243.4	571.1 ± 243.9
			6 SD male rats	Peimine, 4.25 g/kg, ig	6.2 ± 1.9	2.9 ± 1.7	41.5 ± 20.1	374.1 ± 186.2	57.6 ± 21.6	662.4 ± 277.9	665.3 ± 213.3
				Peiminine, 4.25 g/kg, ig	5.1 ± 1.4	3.0 ± 1.4	10.5 ± 2.6	92.2 ± 55.1	135.6 ± 40.2	1965.5 ± 433.3	1969.6 ± 433.5
Tong, 2016 [49]	In vivo	UHPLC-MS/MS; DAS 2.0 package	6 SD male rats; 6 female SD rats	Peimine, 0.45 g/kg, ig	2.7 ± 0.5	0.5 ± 0.2	N/A	40.8 ± 17.6	3.7 ± 0.9	10.5 ± 1.7	10.6 ± 1.7
				Peiminine, 0.45 g/kg, ig	1.9 ± 0.8	0.7 ± 0.2	N/A	9.6 ± 5.0	12.6 ± 2.0	37.1 ± 13.7	37.1 ± 13.8
				Peimisine, 0.45 g/kg, ig	4.0 ± 1.0	0.8 ± 0.3	N/A	12.1 ± 4. 9	1.2 ± 0.2	4.6 ± 2.0	4.7 ± 2.1
Xu et al., 2017 [59]	In vivo	UHPLC-MS/MS; DAS 2.0 package	6 male SD rats	Peimine, 20 g/kg, ig	2.3 ±0.8	2.8 ± 1.4	N/A	N/A	74.7 ± 18.2	474.5 ± 143.4	N/A
				Peimisine, 20 g/kg, ig	2.68 ± 0.78	3.3 ± 1.1	N/A	N/A	15.1 ± 2.0	120.7 ± 31.3	N/A

Note: DAS 2.0: Drug and Statistic 2.0; N/A: Not applicable; SD: Sprague Dawley.

## Supplementary Materials

Supplementary materials can be found at https://www.mdpi.com/1422-0067/20/7/1667/s1.
