# Supplementary material for: Fritillariae Thunbergii Bulbus: Traditional Uses, Phytochemistry, Pharmacodynamics, Pharmacokinetics and Toxicity
Source: Int J Mol Sci. 2019 Apr 3;20(7):1667. doi: 10.3390/ijms20071667 (PMC6479889; doi:10.3390/ijms20071667)
Supplement: Supplementary file 1 [file ijms-20-01667-s001.pdf]

Supplementary file 1. Examples of herbal formulas containing Fritillariae Thunbergii Bulbus in classic literature.

Formula Name	Herbal Ingredients	Indications and /or Actions	Source
**Danggui Beimu Kushen Wan** (Chinese Angelica, Fritillaria and Flavescent Sophora Pill)	Dang gui, Zhe bei mu, Ku shen, Hua shi	Difficult urination during pregnancy with normal diet and appetite	Synopsis of Prescriptions of the Golden Chamber (Jin Gui Yao Lue)
**Shen Gong Bi Xie San** (Marvelous Formula to Avoid Evils)	Ge gen, Jiang can, Sheng di, Ma bo, Chan tui, Jin yin hua, Mai dong, Huang qin, Mu tong, Zhe bei mu, Niu Bang zi, Lian qiao	Severe diphtheria	New Compilation of Empirical Prescriptions (Yan Fang Xin Bian)
**Shen Xian Huo Ming Tang** (God Decoction for Sustaining Life)	Long dan cao, Jin yin hua, Huang qin, Sheng di, Tu fu ling, Sheng shi gao, Mu tong, Ma bo, Che qian zi, Zhe bei mu, Chan tui, Jiang can, Qing guo	Severe diphtheria and sore throat due to wind-heat; Purging fire, removing toxin, clearing heat and nourishing Yin	New Compilation of Empirical Prescriptions (Yan Fang Xin Bian)
**Chu Wen Hua Du San** (Eliminate Febrile Diseases and Clear the Toxin Powder)	Ge gen, Jiang can, Sheng di, Shan dou gen, Chan tui, Dong sang ye, Chao zhi zi, Huang qin, Mu tong, Zhe bei mu, Gan cao, Qing guo	Initial stage of diphtheria, tonsillitis and sore throat; releasing the exterior, soothing the throat, transforming phlegm and reducing swelling	New Compilation of Empirical Prescriptions (Yan Fang Xin Bian)
**Qing Xin Di Fei Tang** (Clear the Heart and the Lung Decoction)	Sheng di, Zhe bei mu, Huang bai, Mai dong, Zhi mu, Tian hua fen, Gan cao, Huang qin, Jiang can, Tian dong	Late stage of diphtheria; clearing residual fire and toxicity	New Compilation of Empirical Prescriptions (Yan Fang Xin Bian)
**Zhi Ru Yong Fang** (Treat Mastitis Decoction)	Pu gong ying, Zhe bei mu, Gan cao, Chai hu, Tian hua fen, Chen pi, Bai zhi, Jing jie, Jin yin hua, Gua lou	Acute mastitis	Compilation of Empirical Prescriptions (Liang Peng Hui Ji Jing Yan Shen Fang)
**Zhuo Ding Jing Yan Hua**	Shan ci gu, Qing dai, Huang bai, Zhe bei mu,	Black plague	Compilation of Black Plague (Shu

Formula Name	Herbal Ingredients	Indications and /or Actions	Source
**He San** (Eliminate Nodules Powder)	Chi xiao dou		Yi Yue Bian)
**Yi Qi Qing Jin Tang** (Augment the Qi and Clear the Lung Decoction)	Jie geng, Huang qin, Zhe bei mu, Mai dong, Niu bang zi, Ren shen, Fu ling, Chen pi, Shan zhi zi, Bo he, Gan cao, Zi su ye, Zhu ye	Tumors in throat; clearing heat and expelling wind, moistening the Lung and tonifying Qi	Golden Mirror of Medicine (Yi Zong Jin Jian)
**Guang Bi Shu Zhan Tang** (Throat Tuberculosis Decoction)	Sheng di, Zhe bei mu, Xuan shen, Gan cao, Niu bang zi, Tian hua fen, She gan, Lian qiao, Jiang can, Zhu ye	Tuberculosis in throat; clearing heat, nourishing Yin, transforming phlegm and soothing throat	Golden Mirror of Medicine (Yi Zong Jin Jian)
**Qing Jin Ning Sou Tang** (Clear the Lung and Stop Cough Decoction)	Ju hong, Qian hu, Gan cao, Xing ren, Sang bai pi, Huang lian, Gua lou zi, Jie geng, Zhe bei mu	Eruption stage of measles with cough	Golden Mirror of Medicine (Yi Zong Jin Jian)
**Xiao Lei Wan** (Eliminate Scrofula Pill)	Mu li, Huang qi, San leng, E zhu, Xue jie, Ru xiang, Mo yao, Long dan cao, Xuan shen, Zhe bei mu	Scrofula; transforming phlegm, softening hardness and dissipating nodules	Integration of Traditional Chinese Medicine and Western Medicine (Yi Xue Zhong Zhong Can Xi Lu)
**Lian Yin Xie Gan Tang** (Astringe Yin and Drain the Liver Decoction)	Bai shao, Tian hua fen, She gan, Zhe bei mu, Shi liu	Pharyngitis; stopping sweating, generating body fluids, and soothing throat	Integration of Traditional Chinese Medicine and Western Medicine (Yi Xue Zhong Zhong Can Xi Lu)
**Xiao Du Yin** (Eliminate Toxin Decoction)	Qing pi, Bai zhi, Dang gui, Chai hu, Zhe bei mu, Jiang can, Tian hua fen, Jin yin hua, Gan cao	Initial stage of acute mastitis	Mirror of Gynecology (Fu Ke Bing Jian)
**Hai Fu Tang** (Intestinal Abscess	Ru xiang, Mo yao, Zhe bei mu, Fu ling, Huang qi, Zhi gan cao	Recovery stage of intestinal abscess; invigorating blood circulation, relieving	Comments on Obstetrics (Ping Zhu Chan Ke Xin Fa)

Formula Name	Herbal Ingredients	Indications and /or Actions	Source
Decoction)		pain, reducing swelling and generating flesh	
**Chai Hu Yin** (Bupleurum Decoction)	Chi shao, Chai hu, Huang lian , Ban xia, Jie geng, Long dan cao, Xia ku cao, Zhe bei mu, Huang qin, Gan cao	Infantile convulsions	Guidelines for Pediatrics (You Ke Zhi Nan)
**Xing Su Yin** (Apricot Kernel and Perilla Leaf Decoction)	Xing ren, Zi su ye, Qian hu, Jie geng, Zhi ke, Sang bai pi, Huang qin, Gan cao, Mai dong, Zhe bei mu, Ju hong, Sheng jiang	Common cold due to wind-heat	Guidelines for Pediatrics (You Ke Zhi Nan)
**Tuo Li Pai Nong Tang** (Support the Interior and Drain Pus Decoction)	Dang gui, Bai shao, Ren shen, Bai zhu, Fu ling, Lian qiao, Jin yin hua, Zhe bei mu, Huang qi, Chen pi, Rou gui, Jie geng, Niu xi, Bai zhi, Gan cao, Sheng jiang	Pus formation stage of carbuncle in occipital region; expelling pus and reducing swelling	Essential Rhymes and Experiential Therapy of External Medicine (Wai Ke Xin Fa Yao Jue)
**Su Qian Tang** (Fructus Perillae and Root of Whiteflower Hogfennel Decoction)	Su zi, Qian hu, Chi shao, Gan cao, Jie geng, Xuan shen, Lian qiao, Zhe beu mu	Diphtheria	Life-saving Manual of Diangosis and Treatment of External Diseases (Wai Ke Zheng Zhi Quan Sheng Ji)
**Bai Zhi San** (Angelica Dahurica Powder)	Ru xiang, Mo yao, Bai zhi, Zhe bei mu, Dang gui	Acute mastitis	Life-saving Manual of Diangosis and Treatment of External Diseases (Wai Ke Zheng Zhi Quan Sheng Ji)
**Ren Shen Bai Du San** (Ginseng Powder to Overcome Toxin)	Xi yang shen, Fang feng, Bai zhi, Zhe bei mu, Jie geng, Jin yin hua, Jiang can, Niu bang zi, Jing jie, Ren zhong huang, Chan tui, Zao jiao ci	Mild diphtheria due to heat	Life-saving Manual of Diphtheria (Bai Hou Quan Sheng Ji)
**Xian Fang Huo Ming Yin**	Jin yin hua, Bei sha shen, Dang gui wei, Bai	Initial stage of any furuncles, carbuncles	Experience in Laryngology (Hou

Formula Name	Herbal Ingredients	Indications and /or Actions	Source
(Sublime Formula for Sustaining Life)	zhi, Chen pi, Gan cao, Zhe bei mu, Tian hua fen, Chuan shan jia, Zao jiao ci, Chi shao, Bai mao gen, Can jian, Ru xiang, Mo yao	and abscesses; clearing heat, removing toxin, reducing swelling, dissipating nodules, invigorating blood circulation and relieving pain	Ke Xin Fa)
**Fang Feng San Jie Tang** (Divaricate Saposhniovia Decoction to Dissipate Nodules)	Bai zhi, Huang qin, Fang feng, Xuan shen, Jie geng, Qian hu, Chen pi, Chi shao, Zhe bei mu, Cang zhu, Tian hua fen	Chalazion	Essential Rhymes and Experiential Therapy of Ophthalmology (Yan Ke Xin Fa Yao Jue)

Note: Herbal ingredients are listed as Chinese pinyin name as per the nomenclature list of commonly used Chinese herbal medicines published by the Chinese Medicine Board of Australia [11]
